# An Elevated Neutrophil-to-Lymphocyte Ratio Predicts In-Hospital Mortality in Stroke Patients: A Prospective Cohort Study

**DOI:** 10.7759/cureus.31372

**Published:** 2022-11-11

**Authors:** Medha Sharath, Ramya B Siddegowda, Ashwini Lonimath, Janardhan D Cheluvaiah

**Affiliations:** 1 Internal Medicine, Bangalore Medical College and Research Institute, Bangalore, IND; 2 Pathology, Bangalore Medical College and Research Institute, Bangalore, IND; 3 Community Medicine, Bangalore Medical College and Research Institute, Bangalore, IND; 4 Neurology, Bangalore Medical College and Research Institute, Bangalore, IND

**Keywords:** neutrophil-to-lymphocyte ratio (nlr), in-hospital mortality, prognosis, inflammatory marker, stroke

## Abstract

Introduction

Cerebrovascular accidents or strokes are a major cause of mortality and morbidity in today's world. Post-stroke disabilities like paralysis, dementia, etc., can affect the quality of life of patients as well as their families. A combined increase in neutrophils and a decrease in lymphocytes during inflammation in stroke manifests as an elevated neutrophil-to-lymphocyte ratio (NLR), thereby indicating the severity of neural damage.

Aim

We aimed to determine if an elevated NLR observed on the day of hospital admission can predict a higher risk of in-hospital mortality in stroke patients. Confirmatory results could aid in developing risk stratification for management, ultimately improving clinical and functional outcomes.

Materials and methods

Sixty stroke patients were monitored throughout their hospital stay in this prospective cohort study. NLR was calculated at admission using routine complete blood counts. The data were analyzed using SPSS Software v23.0 (IBM Corp., Armonk, NY). An unpaired t-test was used to compare the means between the two groups. Categorical data were analyzed using the chi-square test. The receiver operating curve (ROC) was plotted and used to ascertain if a cut-off value of NLR could be obtained to predict in-hospital mortality in stroke patients. P values <0.05 were considered statistically significant.

Results

About 23.3% (n=14) of the patients died during their hospital stay, with no significant differences between the survivor and death cohorts in terms of comorbidities like diabetes and hypertension. The mean NLR calculated within 24 hours of hospital admission in patients who died (NLR=8.47 (standard deviation (SD)=4.67)) was significantly higher (p=0.009) than in those who survived (NLR=5.84 (SD=2.62)). Upon ROC analysis, patients with NLR >6.03 on the day of admission demonstrated a higher risk of in-hospital mortality (p=0.015 (95% CI: 0.577-0.855)). An area under the curve (AUC) of 0.72 with a sensitivity of 92.86% and a specificity of 54.35% was obtained.

Conclusions

Elevated NLR (cut-off >6.03) obtained within 24 hours of hospital admission is an indicator of a higher risk of in-hospital mortality in stroke patients. Hence, patients presenting with a high NLR at admission can be prioritized for personalized targeted treatment, potentially reducing mortality and post-stroke complications.

## Introduction

Cerebrovascular accident or stroke is the sudden onset of the neurological deficit from a vascular mechanism; 85% are due to vascular ischemia, while 15% are due to primary hemorrhage. Stroke is the third leading cause of death in the world and the most common cause of neurological disease in adults. Additionally, it is the third leading cause of disability-adjusted life-years (DALYs) worldwide [1].

Although the mortality rate due to stroke has declined in recent years, the rate of incidence of stroke has increased in middle- and lower-income countries due to health and demographic transitions [2,3]. Major manifestations in stroke survivors include paralysis, post-stroke-associated infections, dementia, speech and cognitive impairment, and emotional disturbances due to loss of independence and identity. Caregivers of disabled stroke survivors also face financial and emotional anxiety since most of the survivors of stroke have lingering functional impairments and require help to complete day-to-day activities [4]. The world's growing elderly distribution will contribute to a large increase in the burden of stroke-related adverse outcomes in the approaching years [2].

Inflammation has a crucial role in the pathophysiology of stroke. Inflammation occurs when stagnant blood flow due to ischemic or hemorrhagic lesions results in the release and accumulation of pro-inflammatory mediators, which lead to the migration of neutrophils to the area of stroke. A decrease in lymphocyte count occurs due to lymphocyte adhesion to endothelial cells during inflammation and also due to lymphocyte migration to inflamed tissues [5].

Thus, the combined increase in neutrophils and decrease in lymphocytes during inflammation manifests as an increased neutrophil-to-lymphocyte ratio (NLR). The aim of this study was to determine if the calculated NLR has any significant relation with the short-term in-hospital mortality rate of stroke patients. Previous studies have demonstrated a relation between elevated NLR calculated at the time of admission and increased in-hospital and three-month mortality rates. However, this study tries to separately determine if there is any statistically significant relation between in-hospital mortality of stroke patients; and between NLR calculated on the day of admission and NLR calculated on day three of hospital stay.

## Materials and methods

Study design

This prospective cohort study was performed in a high-volume tertiary hospital in a dense metropolitan city from July 2021 to May 2022. Based on a previous study by Gokhan et al. [6], the mortality rate of patients with stroke is 11.95. The sample size was calculated by using the formula: 

$\displaystyle n = 4pq/d^{2}$,

where p=proportion=11.95, q=100-p, and d=absolute precision=10. Substituting the values, the sample size was n=42 patients. The subjects of this cohort study were 60 stroke patients admitted to the hospital, whose diagnoses were confirmed by CT scan or MRI.

Inclusion criteria

Patients who were brought to the emergency department with a clinical history of cerebrovascular accident confirmed by CT scan or MRI, and who were admitted to the hospital were included in this study. Patients with both ischemic and hemorrhagic strokes were included in this study. The mean duration from stroke onset to time of admission was 11.28 hours (SD=4.6).

Exclusion criteria

Patients with active infections like HIV and tuberculosis; inflammatory diseases (e.g., malignancy, chronic liver, or renal disease); autoimmune diseases (e.g., rheumatoid arthritis); patients who had undergone recent surgery; patients on immunosuppressive drugs; patients with incomplete data; patients who did not consent to participate in the study; and patients currently having an active SARS-CoV-2 infection were excluded.

Methodology

The National Institutes of Health Stroke Scale (NIHSS) score was calculated at the time of admission. NIHSS score of ≤8 was determined to be a mild stroke, while an NIHSS score of >8 was determined to be a severe stroke. Two samples of whole blood (two ml each) were collected from each patient and analyzed within one hour of collection. The first sample was collected at the time of admission or within 24 hours of admission. The second sample was collected on the third day of the hospital stay. Analysis of the blood samples was done in an automated blood cell counting machine (Beckman Coulter 780, Beckman Coulter Inc., Bangalore) based on the Coulter principle. A complete blood count was obtained. The patients were observed for the entire duration of their hospital stay (mean=7 days) to assess the short-term mortality rate. 

Statistical analysis

Neutrophil-to-lymphocyte ratios (absolute neutrophil counts divided by absolute lymphocyte counts) were calculated manually from complete blood count reports of the patient and compiled in a Microsoft Excel Sheet (Microsoft Corp., Redmond, NY). Continuous variables were presented as mean ± standard deviation (SD), and categorical variables were presented in frequencies and percentages. The data were analyzed using SPSS Software v23.0 (IBM Corp., Armonk, NY). An unpaired t-test was used to compare the means between the two groups. Categorical data were analyzed using the chi-square test. The receiver operating curve (ROC) was plotted and used to ascertain if a cut-off value of NLR could be obtained to predict in-hospital mortality in stroke patients. P values <0.05 were considered statistically significant.

Ethical approval

Institutional ethical clearance was obtained from the Ethics Committee of the Bangalore Medical College and Research Institute (approval ID: BMCRI/PS/254/2020-21). All patients participating in this study were informed in detail of the purpose of the study undertaken, and informed consent was obtained from all patients.

## Results

This prospective cohort study involved a total of 60 stroke patients; 46 (76.6%) patients with acute ischemic stroke and 14 (23.3%) patients with acute hemorrhagic stroke, confirmed through CT scan. Out of the 60 patients, 46 (76.6%) patients survived the hospital stay and were discharged within a week, while 14 (23.3%) patients died within the first week of admission to the hospital. The median baseline NIHSS score was 18 (8-27) among patients who died. The mean age of the patients who survived was 61.06 years (SD=11.24) and that of those who died was 71 years (SD=10.64). Among the 46 patients who survived, 22 (47.8%) were female. Among the 14 patients who died, seven (50%) were female. None of the patients had a previous history of stroke or any other vascular event. All the patients tested negative for SARS-CoV-2 through reverse transcription polymerase chain reaction (RTPCR). About 91.3% of the patients who survived (n=46) and 85.7% of the patients who died (n=14) had uncontrolled diabetes mellitus (HbA1c greater than 7%). Chi-square test was performed and a p-value of 0.54 was obtained. About 95.6% of the patients who survived (n=46) and 85.7% of the patients who died (n=14) had uncontrolled hypertension (systolic BP>140 mm Hg). A P-value of 0.2 was obtained using the chi-square test. Hence, it can be concluded that in this study, there were no statistically significant differences found between the survivor and death cohorts in terms of comorbidities like diabetes and hypertension. Table 1 details the patients' characteristics.

**Table 1:** Baseline characteristics of the study population. NIHSS: National Institutes of Health Stroke Scale, SD: standard deviation.

Baseline characteristics of the study population		
Variable	n=60; SD	
	Patients who survived hospital stay (mean=7 days); n=46 (76.6%)	Patients who died within seven days of hospital stay; n=14 (23.3%)
Mean age in years, y, SD	61.06 (SD=11.24)	71 (SD=10.64)
Median baseline NIHSS score, n (range)	11 (4-25)	18 (8-27)
Women, n (%)	22 (47.8%)	7 (50%)
Diabetes mellitus, n (%)	46 (91.3%)	14 (85.7%)
Hypertension, n (%)	46 (95.6%)	14 (85.7%)

The data were analyzed using SPSS Software v23.0. An unpaired t-test was used to compare the means between the two groups. It was observed that the difference between the mean neutrophil-to-lymphocyte ratio (NLR), calculated within 24 hours of admission, in the patients who survived and in those who died was statistically significant. Table 2 shows the NLR calculated within 24 hours of admission (p value=0.0092).

**Table 2:** Table showing neutrophil-to-lymphocyte ratio (NLR) calculated within 24 hours of admission. By conventional criteria, this result is statistically significant.

Outcome	Neutrophil-to-lymphocyte ratio: unpaired t-test	
	Mean	Standard deviation (SD)
Alive, n=46	5.84	2.62
Dead, n=14	8.47	4.67
p-value=0.0092		

However, in this study, the difference between the mean NLR, calculated on day three of hospital stay, in the patients who survived and in those who died was not statistically significant. Table 3 shows NLR at 72 hours of admission (p value=0.91).

**Table 3:** Table showing the neutrophil-to-lymphocyte ratio (NLR) calculated at 72 hours of admission. By conventional criteria, this result is not statistically significant.

Outcome	Neutrophil-to-lymphocyte ratio: unpaired t-test	
	Mean	Standard deviation (SD)
Alive, n=46	5.56	2.7
Dead, n=14	5.66	3.52
p value=0.91		

**Figure 1:**
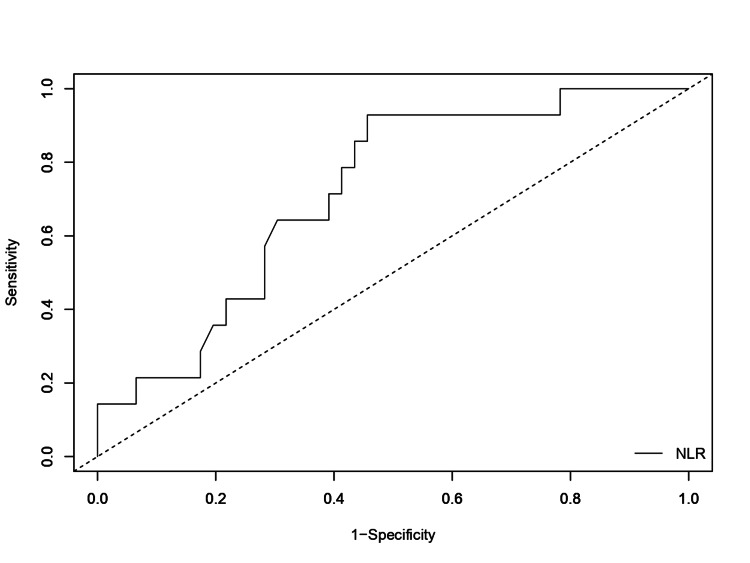
Plotting of the receiver operating characteristic curve yielded an NLR cut-off value of 6.03 and an AUC of 0.72, with a sensitivity of 92.86% and a specificity of 54.35% (p=0.015 (95% CI: 0.577-0.855)). AUC: area under the curve, NLR: neutrophil-to-lymphocyte ratio.

A receiver operating characteristic curve was plotted and analyzed to determine if NLR calculated on the day of admission could predict the mortality of stroke patients (Figure 1). It yielded an NLR cut-off value of 6.03 with an area under the curve (AUC) of 0.72, a sensitivity of 92.86%, and a specificity of 54.35%. The prognostic value of NLR measured on the day of admission to predict in-hospital mortality was statistically significant (p=0.015 (95% CI: 0.577-0.855)). 

## Discussion

Neutrophil-to-lymphocyte ratio (NLR) is a reliable marker of inflammation [7]. Inflammation plays a vital role in the evolution of stroke and its recovery. Pro-inflammatory cytokines, especially IL-8, help in recruiting neutrophils during the inflammatory response [5]. These neutrophils then release various pro-inflammatory cytokines like adhesion molecules (e.g., P-selectin), cytokines (e.g., IL-1, IL-6), chemokines (e.g., CCL2), proteases (e.g., matrix metalloproteinase-9) and free oxygen radicals, which damage neurons and lead to the release of damage-associated molecular patterns (DAMPs) from the injured brain tissue [8,9]. In addition, the thrombus formed in an ischemic stroke can also be further propagated by the activation of platelets and neutrophil extracellular traps [10]. Breakdown of the blood-brain barrier is induced by lytic enzymes released by neutrophils. This is linked to a greater risk of hemorrhagic transformation in ischemic stroke [8,10]. Pathologic cerebral edema also develops as a result of raised blood-brain barrier permeability within 24 to 48 hours in ischemic stroke or within 24 hours in hemorrhagic stroke [8]. This corresponds to a poorer prognosis and unfavorable clinical and functional outcomes.

The role of lymphocytes in the pathogenesis of ischemic stroke is still controversial. While some studies suggest that lymphocytes have a beneficial effect by promoting the resolution of inflammation, other studies report that the production of pro-inflammatory cytokines from lymphocytes further increases damage to neural tissue [7,8]. However, lymphocyte counts have categorically been reported to be lower than baseline values in stroke patients [7]. A reason for this is hypothesized to be the release of glucocorticoids by the hypothalamic-pituitary axis, which produces an anti-inflammatory response and decreases lymphocyte counts [8]. A decrease in lymphocyte count is also due to lymphocyte adhesion to endothelial cells during inflammation and lymphocyte migration to inflamed tissues [5]. Acute brain injury also inactivates the lymphocytes, which markedly impairs the immune system [11]. This leads to an increased occurrence of in-hospital infections after stroke and may worsen the clinical outcome. It may also deteriorate the clinical and functional outcome of the patient by increasing the metabolic demands of the neural tissue, promoting hyperthermia and leading to the development of metabolic acidosis and hypoxia [12].

Ultimately, this combined increase in neutrophils and decrease in lymphocytes during inflammation manifests as an increased NLR. NLR has been established to be higher in patients with ischemic or hemorrhagic stroke compared to patients with transient ischemic attacks or in the control population [7,8]. Elevated NLR values in patients with ischemic stroke have been linked to a higher 30-day mortality rate and an extended duration of hospital stays [8,13]. A meta-analysis of eight studies involving 3855 patients, conducted in 2017, reported that a high cut-off value of NLR (>5) could predict 90-day mortality in ischemic stroke [14]. Another study conducted in Australia determined that the NLR measured in the first 72 hours of admission was significantly elevated in stroke patients who died during their hospital stay compared to those who survived [10]. A higher NLR is associated with a greater chance of developing recurring episodes of ischemic stroke after the initial acute ischemic stroke [7]. However, the mortality rate is also influenced by other factors, such as age, underlying vascular abnormalities, history of myocardial infarction, and baseline National Institute of Health stroke scale (NIHSS) and Glasgow Coma Scale assessments [10,14].

Song et al. found that raised NLR values in patients with hemorrhagic stroke (both subarachnoid hemorrhage and intracranial hemorrhage) were only found to be linked to an adverse effect on mortality but not functional outcome [8]; however, another prospective study of 855 patients with intracranial hemorrhage established that a high NLR (>8.5) was not only associated with higher in-hospital and 30-day mortality rates but also linked to a larger hematoma volume and a poor functional outcome due to a higher frequency of complications like pneumonia or sepsis [15]. A previous study established that an NLR ≥5.79 was strongly associated with an increased risk of developing infections within seven days of experiencing a stroke [16]. Giede-Jeppe et al. also concluded that a higher frequency of infections like pneumonia and sepsis was observed in patients with NLR >8.5 [15]. Tokgoz et al. determined that although a high NLR at admission was associated with a greater rate of mortality in acute hemorrhagic strokes [17], the test was less specific and sensitive compared to the relation between a higher NLR and short-term mortality in patients with acute ischemic stroke. Raised baseline NLR levels in patients with ischemic stroke were associated with a greater risk of hemorrhagic transformation after thrombolysis [8]. This may be due to the damage to the blood-brain barrier integrity and cerebral vessels by neutrophils or the antithrombotic effect of thrombolysis therapy. An elevated neutrophil count was also found to be an independent predictor of poor outcome at 90 days despite recanalization. Thus, post-stroke NLR may serve as a marker for patients who might need a hemicraniectomy for large infarcts in spite of successful recanalization [8]. A combined index of NLRs and platelet-to-lymphocyte ratios (PLRs), rather than either alone, was also found to be a meaningful marker of early detection of depression six months after stroke [18].

This study confirmed previous findings, as the mean NLR calculated within 24 hours of hospitalization was higher among patients who died than among those who survived, and this difference was statistically significant. However, the difference in NLR calculated on day three of hospital stay between the patients who survived and those who died were not statistically significant in this study. Upon ROC analysis, an NLR cut-off value of 6.03, with an area under the curve (AUC) of 0.72, a sensitivity of 92.86%, and a specificity of 54.35% was obtained. The prognostic value of NLR measured within 24 hours of admission to predict in-hospital mortality was statistically significant (p=0.015 (95% CI: 0.577 to 0.855)). In our study, 59% (n=13) of the patients who had an NLR >6.03 on the day of admission died within seven days of their hospital stay.

Conversely, a study conducted by Wang et al. found no relation between the 30-day mortality rate and NLR calculated at the time of admission in patients with acute hemorrhagic stroke [19]. Lashin et al. also did not find any significant association between NLR and the 30-day outcome in acute ischemic stroke patients [20].

The NLR has been hypothesized to increase within twelve hours of the onset of stroke and attain the maximum value by 24-72 hours. Neutrophil counts then fall rapidly, leading to a corresponding decline in NLR [10]. This decrease in neutrophils during stroke recovery may help limit blood-brain barrier damage by decreasing the amount of pro-inflammatory cytokines released, thereby improving the prognosis of stroke patients. Recanalization of vessel occlusions also decreases NLR by re-establishing blood flow and bringing lymphocytes to the site of stroke, thereby limiting damage by the mediators released by neutrophils [8]. This was corroborated in this study, where the mean NLR calculated on day three of the hospital stay was lower than the mean NLR values obtained on the day of admission. The mean NLR on the day of admission in patients who survived was 5.84 (SD=2.62), which decreased to 5.56 (SD=2.70) on day three. The mean NLR on the day of admission in those who died was 8.47 (SD=4.67), which decreased to 5.66 (SD=3.52) on day three of the hospital stay. 

Strokes are diagnosed clinically by evaluating the neurologic deficits observed during the clinical examination of the patient and confirmed by CT or MRI. However, in developing countries, the unavailability of CT or MRI facilities in small rural hospitals impedes the diagnosis of stroke patients, slowing their accessibility to treatment and potentially worsening the outcome. These drawbacks necessitate the development of a convenient test that can provide an initial indication of the patient's prognosis. NLR is one such marker that is routinely measured as part of complete blood counts. It has previously been used to identify inflammation in the clinical setting [8]. Although it has limited sensitivity and specificity, it still provides a reliable method to predict the mortality and prognosis of stroke patients [10].

As a result of these observations, anti-inflammatory therapy has been proposed to potentially improve prognosis and functional outcomes, in addition to the standard intravascular thrombolysis and reperfusion therapy given for ischemic strokes [8]. A study on animal models has shown that selectively inhibiting neutrophils and neutrophil-derived matrix metalloproteinases after an attack of stroke decreased the microglial and macrophage response and improved recovery [21]. However, another study found that the use of anti-neutrophilic factors did not improve recovery in patients with acute ischemic stroke [22]. This could be due to the fact that some molecules might help improve neural tissue healing and recovery during inflammatory responses [7]. The presence of underlying inflammation due to pre-existing diseases might also act as a confounder. Thus, further research must be conducted to establish whether anti-inflammatory therapy significantly improves the clinical and functional outcomes of stroke patients.

This study does have some limitations. A study with a higher power, consisting of a larger sample population, can provide more meaningful insights into the relation between the neutrophil-to-lymphocyte ratio and the prognosis of stroke patients. Although this study did not find any statistically significant differences between the survivor and death cohorts in terms of comorbidities like diabetes and hypertension, studies with larger sample sizes might provide a different outcome. In addition, all the data for this study was collected from a single high-density tertiary hospital; hence, the possibility of selection bias cannot be ruled out. Finally, since patients with both ischemic and hemorrhagic strokes were included in this study, some heterogeneity might be present.

## Conclusions

This study has found that the mean NLR calculated within 24 hours of hospitalization was higher among patients who died than among those who survived. In addition, a cut-off value of NLR >6.03 has been shown to have a predictive value on in-hospital mortality. Since this study demonstrated that an elevated NLR calculated on the day of admission had a predictive value on in-hospital mortality but an NLR calculated on day three did not, importance should be given to the timely collection of blood samples. If calculating NLR at the early stages of stroke-induced inflammation can help predict a poor prognosis, patients presenting with a high NLR at admission can be prioritized for targeted treatment, thereby potentially reducing mortality and post-stroke complications. It can also help families of stroke victims prepare for adverse outcomes in the case of a poor prognosis, improving the quality of end-of-life care provided to patients.

## References

[1] Murray CJ, Vos T, Lozano R (2012). Disability-adjusted life years (DALYs) for 291 diseases and injuries in 21 regions, 1990-2010: a systematic analysis for the global burden of disease study 2010. Lancet.

[2] Kim AS, Cahill E, Cheng NT (2015). Global stroke belt: geographic variation in stroke burden worldwide. Stroke.

[3] Feigin VL, Lawes CM, Bennett DA, Barker-Collo SL, Parag V (2009). Worldwide stroke incidence and early case fatality reported in 56 population-based studies: a systematic review. Lancet Neurol.

[4] Murray J, Ashworth R, Forster A, Young J (2003). Developing a primary care-based stroke service: a review of the qualitative literature. Br J Gen Pract.

[5] Rentauli M, Kurniawan LB, Muhadi D (2019). Diagnostic value of neutrophil-lymphocyte ratio to differentiate ischemic and hemorrhagic stroke. J Clin Pathol Med Lab.

[6] Gökhan S, Ozhasenekler A, Mansur Durgun H, Akil E, Ustündag M, Orak M (2013). Neutrophil lymphocyte ratios in stroke subtypes and transient ischemic attack. Eur Rev Med Pharmacol Sci.

[7] Xue J, Huang W, Chen X, Li Q, Cai Z, Yu T, Shao B (2017). Neutrophil-to-lymphocyte ratio is a prognostic marker in acute ischemic stroke. J Stroke Cerebrovasc Dis.

[8] Song SY, Zhao XX, Rajah G (2019). Clinical significance of baseline neutrophil-to-lymphocyte ratio in patients with ischemic stroke or hemorrhagic stroke: an updated meta-analysis. Front Neurol.

[9] Nasr N, Ruidavets JB, Arnal JF, Sie P, Larrue V (2009). Association of neutrophil count with microembolization in patients with symptomatic carotid artery stenosis. Atherosclerosis.

[10] Wijeratne T, Sales C, Karimi L, Jakovljevic M (2021). Elevated neutrophil to lymphocyte ratio predicts in-hospital mortality among stroke patients in a metropolitan hospital in Australia, universal value-added measure in stroke care [PREPRINT]. medRxiv.

[11] Zhang J, Cai L, Song Y (2017). Prognostic role of neutrophil lymphocyte ratio in patients with spontaneous intracerebral hemorrhage. Oncotarget.

[12] Lattanzi S, Cagnetti C, Provinciali L, Silvestrini M (2016). Neutrophil-to-lymphocyte ratio predicts the outcome of acute intracerebral hemorrhage. Stroke.

[13] Vural G, Gümüşyayla S, Akdeniz G (2018). Neutrophil/lymphocyte ratio in stroke patients and its relation with functional recovery. Medeniyet Med J.

[14] Ye Z, Ai X, Fang F, Hu X, Faramand A, You C (2017). The prediction of neutrophil to lymphocyte ratio for outcomes in ischemic stroke. Oncotarget.

[15] Giede-Jeppe A, Bobinger T, Gerner ST (2017). Neutrophil-to-lymphocyte ratio is an independent predictor for in-hospital mortality in spontaneous intracerebral hemorrhage. Cerebrovasc Dis.

[16] He L, Wang J, Wang F, Zhang L, Zhang L, Zhao W (2020). Increased neutrophil-to-lymphocyte ratio predicts the development of post-stroke infections in patients with acute ischemic stroke. BMC Neurol.

[17] Tokgöz S, Uca AU, Poyraz N (2019). The effect of neutrophil lymphocyte ratio on prognosis in acute hemorrhagic stroke: a retrospective study. Austin J Cerebrovasc Dis Stroke.

[18] Hu J, Zhou W, Zhou Z, Han J, Dong W (2020). Elevated neutrophil-to-lymphocyte and platelet-to-lymphocyte ratios predict post-stroke depression with acute ischemic stroke. Exp Ther Med.

[19] Wang F, Hu S, Ding Y, Ju X, Wang L, Lu Q, Wu X (2016). Neutrophil-to-lymphocyte ratio and 30-day mortality in patients with acute intracerebral hemorrhage. J Stroke Cerebrovasc Dis.

[20] Lashin M, Khalil S, Alloush T, Anis S, Fouad M (2020). Neutrophil to lymphocyte ratio in acute ischemic stroke. Neurosci Med.

[21] Wang J, Tsirka SE (2005). Neuroprotection by inhibition of matrix metalloproteinases in a mouse model of intracerebral haemorrhage. Brain.

[22] Krams M, Lees KR, Hacke W, Grieve AP, Orgogozo JM, Ford GA (2003). Acute stroke therapy by inhibition of neutrophils (ASTIN): an adaptive dose-response study of UK-279,276 in acute ischemic stroke. Stroke.

